# Removing Salivary Calculus with Acids

**Published:** 1872-05

**Authors:** 


*Removing Salivary Calculus with Acids.*—On this subject, Dr. H. S. Chase in the *Missouri Dental Journal*, replies to an "eminent man in the profession," who recommends the use of acids for the removal of calculi from the teeth:

"Acids that will remove calculi from the surface of the teeth, will also act upon the tooth's substance itself, whether it be enamel, dentine or cementum. It was said in the discussion that acids would not act on the living bone tissue. If this was so then *decay* of the teeth would never take place. But it is not so. No one is justified in the use of acids for removing oral calculi from the teeth. The advocacy of a contrary doctrine would give license to all the mountebanks and humbugs who follow circus caravans and country fairs, and use sulphuric acid for whitening the teeth, besides leading into error thousands of young practitioners, who look to the older members of the profession for advice and correct practice.

"Its removal should be accomplished by *mechanical* means, instead of chemical. Scalers, having a fine, sharp edge, should be used, of a variety of patterns and sizes. Small excavators and chisels are also useful. Every particle of calculus that can be seen with the aid of glasses should be taken away, particularly that portion at the neck of the tooth. Small particles often are hidden under the edge of the gums; none should be left here. The debris should be washed away with warm water, and the syringe. Finally, every portion of the tooth which has been subjected to the use of sharp instruments, or from which tartar has been removed, should be polished smoothly with pumice stone, and with chalk."

